# Preparation and mechanism study of *Alhagi honey* polysaccharide-aluminum Pickering emulsion adjuvant for improving intestinal mucosal immune function

**DOI:** 10.3389/fimmu.2026.1775276

**Published:** 2026-02-24

**Authors:** En Zhou, Jiubin Du, Yongbin Zhai, Yan Xiao, Guangyan He, Dilinuer Abudureheman, Saifuding Abula, Shengyi Wang, Adelijiang Wusiman

**Affiliations:** 1 College of Veterinary Medicine, Xinjiang Agricultural University, Urumqi, China; 2 Xinjiang Key Laboratory of New Drug Study and Creation for Herbivorous Animals (XJ-KLNDSCHA), Xinjiang Agricultural University, Urumqi, China; 3 Tiankang Biopharmaceutical Co., Ltd, Urumqi, China; 4 Lanzhou Institute of Husbandry and Pharmaceutical Sciences of Chinese Academy of Agricultural Sciences, Lanzhou, China

**Keywords:** *Alhagi honey* polysaccharide, mechanism of action, Pickering emulsions, retinoic acid, vaccine adjuvants

## Abstract

**Introduction:**

Bovine viral diarrhea virus (BVDV) can induce diarrhea and mucosal tissue damage, and in severe cases, may result in fatal. Targeted adjuvants capable of enhancing intestinal IgA antibody responses represent an effective strategy for preventing bovine viral diarrhea.

**Methods:**

In this study, an *Alhagi honey* polysaccharide Pickering emulsion (AHPPE) adjuvant was developed by incorporating *Alhagi* honey polysaccharide (AHP) and retinoic acid (RA) with an aluminium-based adjuvant. Mice were subsequently immunized via intramuscular injection to evaluate the adjuvants' immunostimulatory potential.

**Results:**

The results demonstrate that the particle size of AHPPE is 2133 nm, with excellent dispersion and stability. The encapsulation efficiencies for BVDV and AHP were 66.8% and 73.2%, respectively. AHPPE demonstrated recruitment of antigen-presenting cells at the injection site and activated IgA cells in the duodenum, jejunum, and ileum, inducing to increased IgA expression across multiple intestinal segments. Furthermore, AHPPE significantly induced serum IgG production and elevated levels of cytokines, including IL-4, IL-10, IL-17, IFN-γ, and TNF-α (*P*<0.05). Through sequencing analysis, it was found that IgA production may be induced via Intestinal immune network for IgA production, thereby mediating intestinal mucosal immune responses.

**Conclusion:**

Collectively, these findings indicate that AHPPE adjuvant administered via injection can simultaneously induce effective systemic immune and intestinal mucosal immunity. Therefore, as a novel intestinal-targeted adjuvant, AHPPE shows potential to enhance the specific intestinal mucosal immunity and systemic immunity of BVDV vaccine.

## Graphical Abstract

**Figure:**
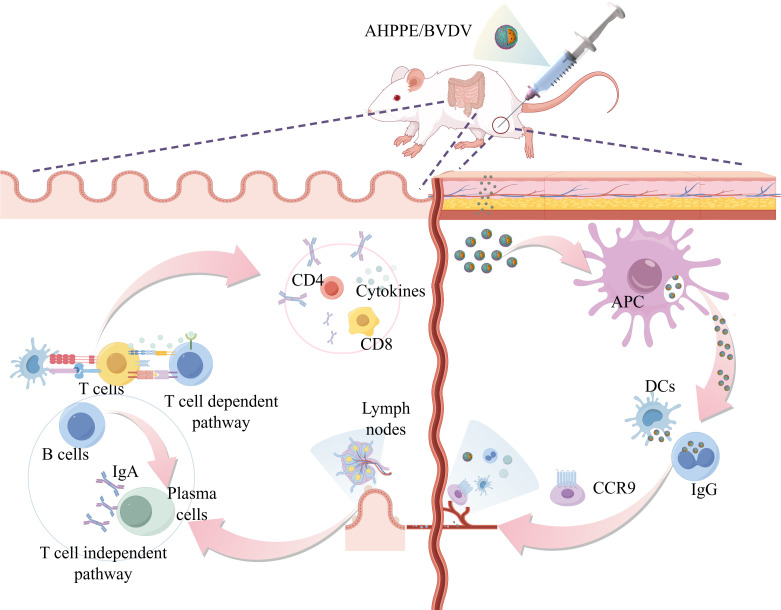
After mixing the *Alhagi* honey polysaccharide Pickering emulsion with the antigen BVDV, and immunising mice through intramuscular injection into the skin, the antigen-presenting cells undergo uptake phagocytosis, recruiting dendritic cells and macrophages, and then homing to the intestine through the action of the intestinal homing receptor CCR9, and then B cell activation in the intestinal lymph nodes via a T cell-dependent route to produce IgA antibodies, and immune cell activation via a non-translocated T cell-dependent route, leading to IgA antibody production; thus inducing systemic and mucosal immunity. Activation of immune cells through a T-cell-dependent route, resulting in the production of IgA antibodies; this in turn induces systemic immunity and intestinal mucosal immunity.

## 1 Introduction

In the past few years, the increasing frequency of disease outbreaks and pandemics has highlighted the critical role of vaccines in public health and disease control (1). Rotavirus as a leading cause of viral diarrhea in livestock, particularly cattle, threat to public health and livestock production (2, 3). Virus-induced diarrhea is a contagious intestinal disease posing a significant threat to public health and livestock production. Immunization remains one of the most effective strategies for the prevention and control of infectious diarrheal diseases (4, 5). The incorporation of adjuvants in vaccine formulations markedly enhance immunogenicity, extend the duration of protective immunity, and reduce both the required vaccine dosage and the number of administrations (6). Within the defense mechanisms, the mucosal system acts as the primary barrier against pathogen entry. Within the gastrointestinal tract, secretory immunoglobulin A (sIgA) plays a critical role in neutralizing pathogens and regulating microbiota homeostasis (7, 8). Animal digestive system diseases including viral gastroenteritis, BVDV, bovine rotavirus and Porcine epidemic diarrhea (PED), need not only improved systemic immunity but also effective intestinal mucosal immunity (9, 10). Therefore, developing a novel vaccine adjuvant with minimal side effects that simultaneously activate both mucosal and systemic immune responses kept a promising approach for improving vaccine efficacy.

Additionally, the selection of emulsion adjuvants demonstrated significant attention because of their direct method and advantageous characteristics, including as the ability to carry multiple drugs with exceptional stability (11, 12). These emulsions are used as carriers, incorporating aluminium-based adjuvants onto the surface of oils such as squalene via ultrasonic assembly. This method produces spherical assembled emulsion particles, in which the oil phase forms the core and the hydrophilic solid carrier constitutes the shell (13, 14). Aluminium adjuvants enhance humoral immune responses by adsorbing antigens, triggering local inflammatory reactions at the injection site, and recruiting and activating immune cells (15–17). However, their capacity to induce cellular and mucosal immunity remains limited, representing a notable shortcoming.

Notably, Picking an emulsion composed of aluminium adjuvant and squalene has been shown to enhance vaccine immunogenicity and protective efficacy, while overcoming the limitations of aluminium adjuvant alone (18). These emulsions can simultaneously induce both cellular and humoral immune responses (16, 19). AHP, derived from *Alhagi Honey*., a medicinal plant native to Xinjiang, has been shown to promote intestinal sIgA secretion, thereby effectively contributing to protecting intestinal mucosal protection and enhancing mucosal immune function (20). However, polysaccharides used as adjuvants typically exhibit a rapid metabolic rate *in vivo*, leading to a short duration of action and limited tissue targeting. A hydrophobic small molecule, all-trans retinoic acid (RA), can activate innate immune cells, enabling DC, T and B lymphocytes to home to intestinal tissues and exert effective intestinal targeting effects (21). Therefore, the incorporation of plant polysaccharides and all-trans retinoic acid into the Pickering emulsion-based adjuvant formulation may provide a synergistic immunomodulatory effect, thereby enhancing both systemic immune responses and intestinal mucosal immunity responses in the body.

In the present research, we propose a new type of Pickering emulsion adjuvant composed of AHP, aluminium adjuvant, squalene, and RA, prepared via phacoemulsification. The physicochemical characteristics of the Pickering emulsion adjuvant were analyzed using a particle size analyzer, laser confocal microscope, and cryo-scanning electron microscopy. Subsequently, *in vivo* experiments were conducted in mice, wherein the Pickering emulsion adjuvant was combined with the BVDV antigen as a vaccine candidate. post-immunization (including IgG and IgA) analysis revealed that this adjuvant formulation significantly enhanced both humoral immunity and mucosal immunity, indicating a synergistic immune-enhancing effect. These findings confirm the effectiveness of the proposed adjuvant approach and provide significant perspectives for the creation of more accessible, efficient, and environmentally friendly vaccine systems.

## 2 Materials and methods

### 2.1 Formulation of AHPPE emulsion

AHPPE was prepared using the ultrasonic emulsification method. First, aluminium (alum) was pretreated by sonication on ice for 1 minute and subsequently mixed with AHP (2.5 mg/mL) to prepare an AHP-alum solution (6 mg/mL), which served as the aqueous phase. RA and squalene, both sourced from Shanghai McLean Biochemistry Science and Technology Co., Ltd., were then used to formulate an RA solution with a concentration of 0.625 mg/mL as the oil phase. The aqueous and oil phases were phacoemulsification on ice for 3 min in a ratio of 11:1 (W: O=V_11_:V_1_) to obtain a uniform AHPPE emulsion.

### 2.2 Morphology and stability of AHPPE emulsion

The morphology of AHPPE was and characterized using scanning electron microscopy and laser confocal microscopy. To evaluate the antigen loading properties of AHPPE, FITC-dextran (Dextran-FITC) (green, Beijing Solaibao Technology Co., Ltd.) was substituted for polysaccharides, and the oil phase was labelled with CY5.5 (red, Beijing Solaibao Technology Co., Ltd.). The morphology and structure of AHPPE were observed under laser confocal microscopy. To evaluate stability, the prepared AHPPE emulsion was stored at 4 °C and 37 °C, and samples were collected at 0 h, 4 d, 40 d, 80 d, and 100 d, respectively, to observe whether the AHPPE was precipitated and delaminated.

### 2.3 Characterization of AHPPE emulsion

A Malvern particle size analyzer was used to assess the particle size. PDI, and zeta potential of AHPPE emulsion over time. Measurements were taken at 4 °C and 37 °C on days 1, 4, 7, 20, 40, 80, and 100 to monitor changes in physical properties and stability.

### 2.4 Efficiency of antigen loading and *in vitro* release of antigen from AHPPE emulsion

To determine antigen loading efficiency, AHPPE was mixed with BVDV antigen and incubated for 15 min, followed by a 1 h resting period. The emulsion was then centrifuged at 12000 rpm for 30 min. The supernatant was collected to determine the concentration of free AHP using the phenol-sulfuric acid method, and free BVDV was quantified using a BCA protein assay kit. These measurements were used to calculate the antigen and polysaccharide loading efficiencies.

### 2.5 Animal immunization

All experimental protocols were authorized by the Animal Welfare Ethics Committee of Xinjiang Agricultural University (Approval No. 2022016). Mice were euthanized by cervical dislocation. BVDV antigen was provided by Tiankang Biopharmaceutical Co., Ltd. AHPPE, PE, and Alum were incubated with BVDV antigen at a 3:1 ratio for 30 minutes to ensure optimal adsorption of the antigen onto the adjuvant surface. A total of 100 six-week-old BALB/c mice were randomly allocated into five groups (n = 20). The mice were intramuscularly injected with 100 μL of AHPPE, PE, Alum, BVDV antigen (20 μg/mL), or PBS (without BVDV antigen). Blood samples were collected from the mice under anesthesia on days 3, 7, 14, and 35. The serum was isolated and stored for further analysis. After euthanasia, visceral organs were harvested for subsequent analysis.

### 2.6 *In vivo* fluorescent section analysis

To evaluate the targeting ability of AHPPE to the intestinal tract, an *in-vivo* fluorescence imaging test was performed. Six-week-old BALB/c mice (n = 15 per group) were divided into four groups: control group (Cy5.5), alum group (Alum/Cy5.5), PE group (PE/Cy5.5), and AHPPE group (AHPPE/Cy5.5). The above four groups were labelled with the fluorescent dye Cy5.5 (25 μg) dissolved in DMSO at an early stage. Mice were injected intramuscularly with 100µL of the respective formulations. At 3 and 10 days post-injection, the intestinal tracts of mice were harvested, fixed in 4% paraformaldehyde in the dark, stained with DAPI, and imaged using the confocal microscope. Quantitative analysis was performed using ImageJ software. The injection sites of mice were collected on day 3 and analyzed by flow cytometry to detect the recruitment effect of the injection sites.

### 2.7 Detection of specific antibodies and cytokine levels

Serum samples were collected on days 3, 7, 14, and 35 post-immunization for the measured of BVDV-specific IgG levels using an indirect ELISA assay. Concurrently, small intestinal tissues were harvested and homogenized for protein quantification using a BCA kit. The levels of BVDV-specific IgA in the small intestine were also measured via ELISA. On day 14, after immunization, serum cytokine levels (TNF-α, IFN-γ, IL-4, IL-10, and IL-17) were detected by an ELISA kit (Shanghai Kexing Trading Co., Ltd).

### 2.8 Injection site recruitment and T-cell activation

On day 14 post-immunization, splenic lymphocytes and injection sites (n = 5) were collected from mice. The injection site and spleen were dissected, minced into small fragments, and homogenized using a tissue grinder. The resulting cell suspension was filtered through a 100-mesh sieve to obtain a single-cell suspension. Stained with the following antibodies: anti-mouse-CD11c-FITC antibody, anti-mouse-CD11b-APC antibody, anti-mouse-CD3e-FITC antibody, anti-mouse-CD4-PE antibody, and anti-mouse-CD8a-APC antibody (All antibodies were commercially obtained from France Abcam Co., Ltd). Flow cytometry analysis was conducted to evaluate immune cell recruitment at the injection site and T-cell differentiation expression.

### 2.9 Immunofluorescence detection of the sIgA

Immunofluorescence analysis was used to determine the expression level of the sIgA protein. Small intestinal tissue sections were co-incubated with specific sIgA antibody and washed with PBS. FITC-labelled secondary antibody was added and incubated at room temperature under light-protected conditions and washed with PBS. DAPI staining solution was added and incubated under light-avoidant conditions and washed with PBS. After washing and treatment with an autofluorescence quencher, sections were rinsed under running water for 10 min. Sections were treated with an anti-fluorescence blocking agent, followed by imaging under a fluorescence microscope and quantitative analysis using ImageJ software.

### 2.10 Transcriptome sequencing

At day 14 post-immunization, ileum samples were collected on ice, immediately flash-frozen in liquid nitrogen, and stored at -80 °C until further processing. The specimens were then transported to Benner Biotech Co., Ltd. under controlled conditions for transcriptomic analysis.

### 2.11 Detection of intestinal mRNA and protein expression levels

At day 14 post-immunization, intestinal RNA was extracted and reverse transcribed according to the instructions (Fuchs Bio); the system was formulated and assayed on the machine according to the instructions for the qPCR reagents (Fuchs Bio), and the results were calculated according to 2^-(ΔΔCt). The gene sequences employed in this study are provided in **Supplementary Table S1** within the **Supplementary Materials**.

For protein expression analysis, Intestinal tissues were excised and fixed in 4% paraformaldehyde, paraffin-embedded, and sectioned. Sections were treated with 3% hydrogen peroxide in methanol for 15 minutes to inhibit endogenous peroxidase activity and then blocked with a 5% bovine serum albumin (BSA) solution. Incubated with primary antibodies overnight at 4 °C, followed by secondary antibodies at 37 °C. DAB chromogenic substrate was used for color development. Sections were dehydrated, seal sheet, and imaged under a microscope. Image analysis was performed using ImageJ software.

### 2.12 Statistical analyses

The data are presented as mean ± SEM. Statistical analysis was performed using one-way ANOVA, followed by Tukey’s test. Statistical significance was considered when the probability value (*P*) was lower as 0.05. *P < 0.05, **P < 0.01, ***P < 0.001, ns: not significant.

## 3 Results

### 3.1 Characterization of AHPPE

**Figure 1:**
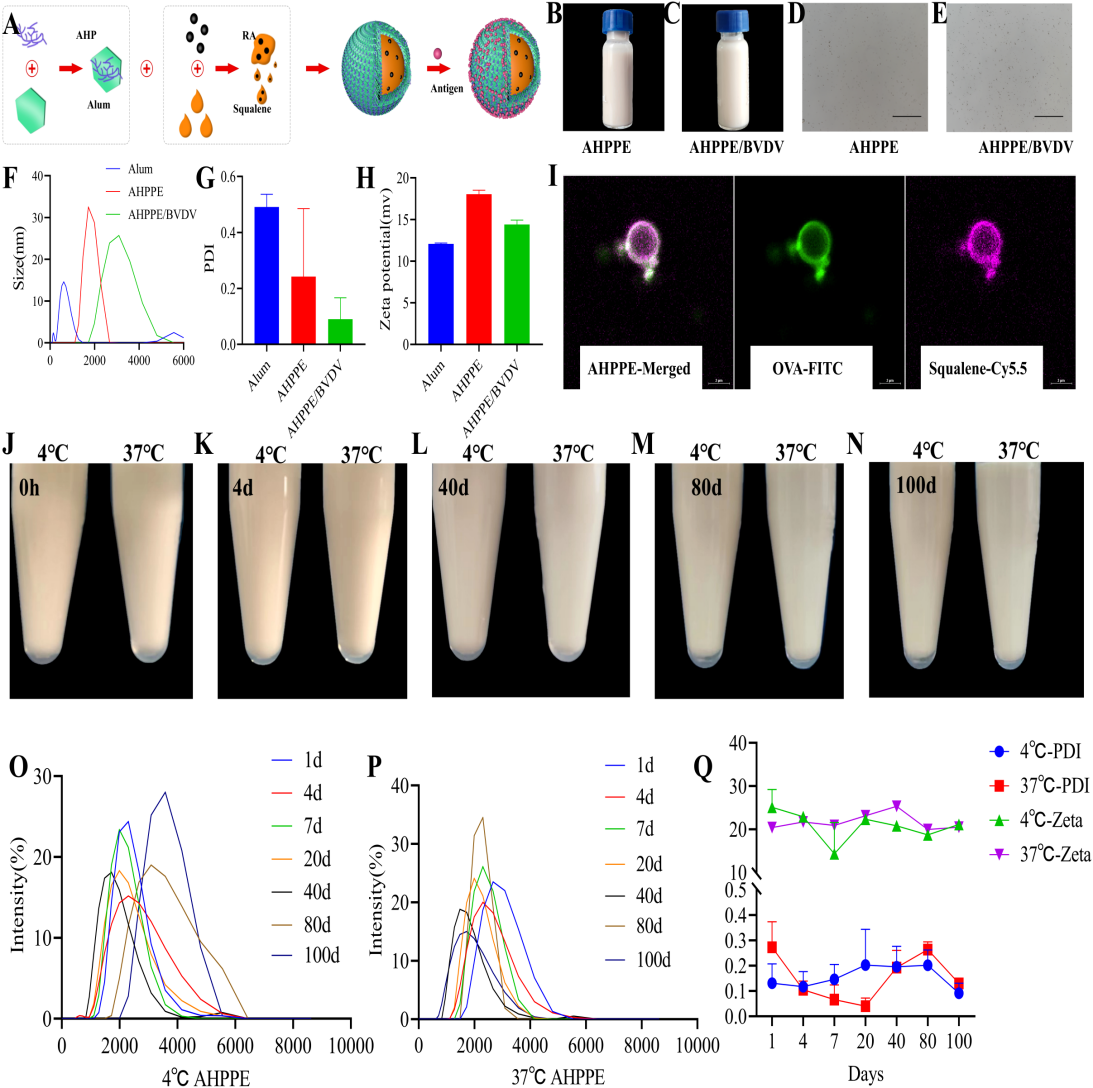
Preparation and representation of AHPPE. **(A)** Preparation diagram of AHPPE, **(B)** Appearance image of AHPPE, **(C)** Appearance image of AHPPE/BVDV, **(D)** Microscopic morphology of AHPPE, **(E)** Microscopic morphology of AHPPE/BVDV, **(F)** Size, **(G)** PDI, **(H)** Zeta potential. The scale bar in the electron microscopic images is 100 μm, **(I)** Confocal microscopy images of AHPPE (purple, Cy5.5; green, Dextran-FITC; scale bar = 2 μm), **(J-N)** stability of AHPPE under various conditions, **(O)** Size at 4 °C, **(P)** Size at 37 °C, **(Q)** PDI and zeta potential of AHPPE under different conditions and data presentation is in mean ± SEM format (n = 3).

AHPPE emulsions were prepared via ultrasonication using RA and squalene as oil phase components, with Alum serving as a stabilizer at the water-oil interface. The resulting emulsions facilitated electrostatic adsorption of antigens onto their surfaces (**Figure 1A**). Electron microscopy revealed that AHPPE appeared as a uniformly distributed, milky white spherical emulsion with homogeneous particles (**Figures 1B–E**). As illustrated in **Figures 1F–H**, the particle size of Alum alone was approximately 610 nm. The average particle size of AHPPE emulsion was about 2133 nm, which increased to approximately 2738 nm upon loading with BVDV antigen (AHPPE/BVDV complex). The PDI of Alum was about 0.5, and the PDI of both AHPPE and AHPPE/BVDV was less than 0.3. Additionally, all formulations displayed a positive zeta potential, facilitating the effective electrostatic adsorption of the negatively charged BVDV antigen. As shown in **Figure 1I**, AHPPE emulsions displayed a spherical morphology with an average particle size of approximately 2000 nm. Fluorescent imaging confirmed that Dextran-FITC was successfully adsorbed onto the surface of the squalene oil core, demonstrating an oil-in-water structure with an internal oil phase and external aqueous phase, typical of oil-in-water adjuvants. In addition, a preliminary evaluation of AHPPE emulsions storage stability was conducted by storing the emulsions at 4 °C and 37 °C for 100 days. As shown in **Figures 1J–N**, no precipitation or phase separation was observed during this period. The particle size distribution remained monodisperse with a single peak profile at both temperatures throughout the 100-day storage, indicating that no significant degradation and polymerization occurred (**Figures 1O, P**). Zeta potential measurements further demonstrated that AHPPE remained positively charged under 4 °C and 37 °C (**Figure 1Q**). The average PDI of AHPPE remained below 0.3 over 100 days (**Figure 1Q**). The AHP and BVDV loading efficiencies of AHPPE/BVDV were 73.2 ± 0.19% and 66.8 ± 2.61%. **Supplementary Figure S1** verified the biological safety of AHPPE, and no pathological infiltration was observed.

### 3.2 Recruitment effect at the injection site

**Figure 2:**
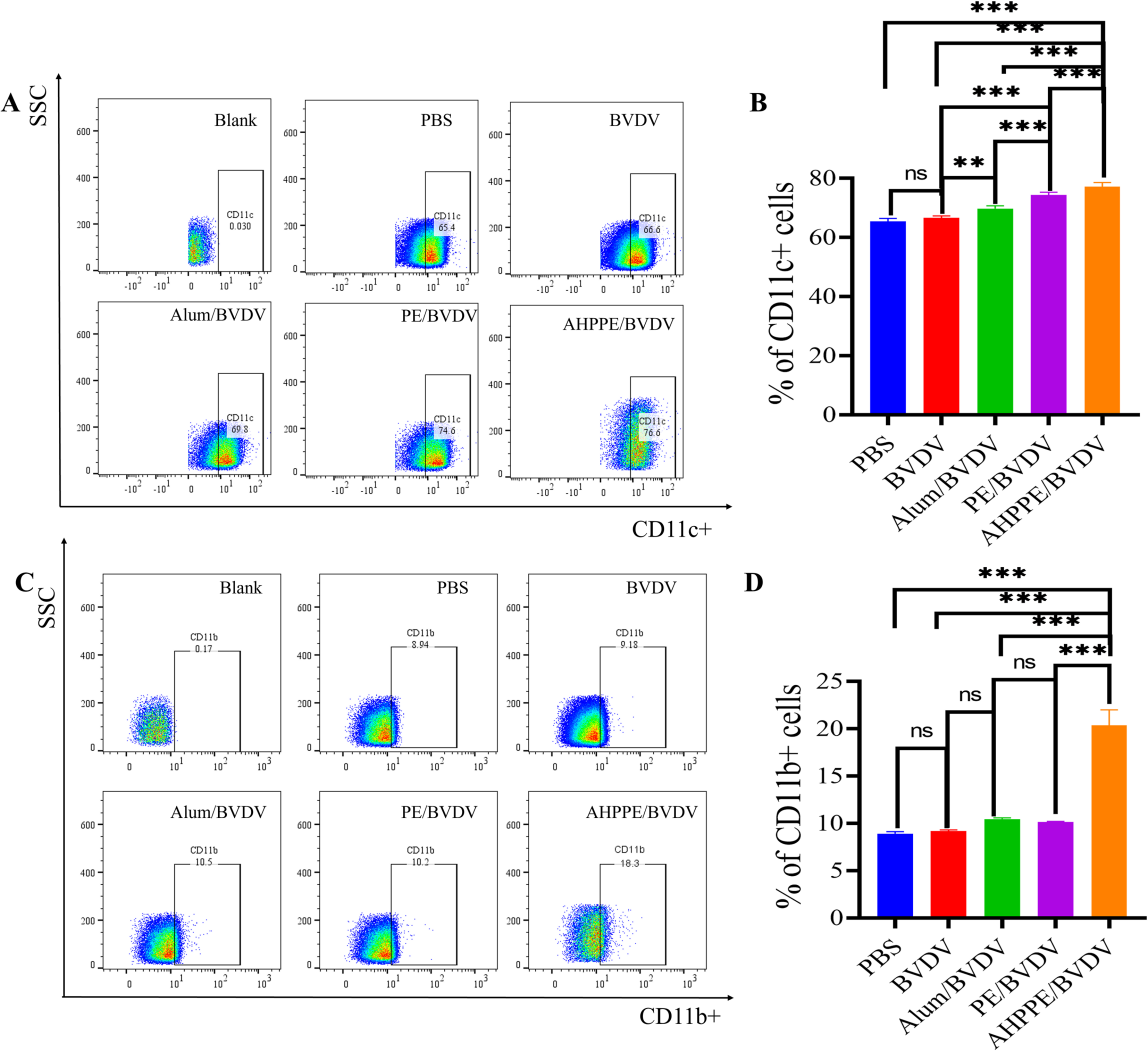
The recruitment effect at the injection site. **(A, C)** Flow type for CD11c and CD11b, **(B, D)** The cell proportions of CD11c and CD11b. Data presentation is in mean ± SEM format (n = 4). *P < 0.05, **P < 0.01, ***P < 0.001, ns, not significant.

Flow cytometry was employed to assess the cell recruitment capacity at the injection site. The number of CD11c^+^ cells was markedly increased in the AHPPE/BVDV group compared to that PBS, BVDV, Alum/BVDV, and PE/BVDV groups (*P* < 0.05)(**Figures 2A, B**). A similar trend was observed for CD11b^+^ cells at the injection site, with significantly greater accumulation in the AHPPE/BVDV group with the other groups (*P* < 0.05)(**Figures 2C, D**).

### 3.3 *In vivo* fluorescent for AHPPE

**Figure 3:**
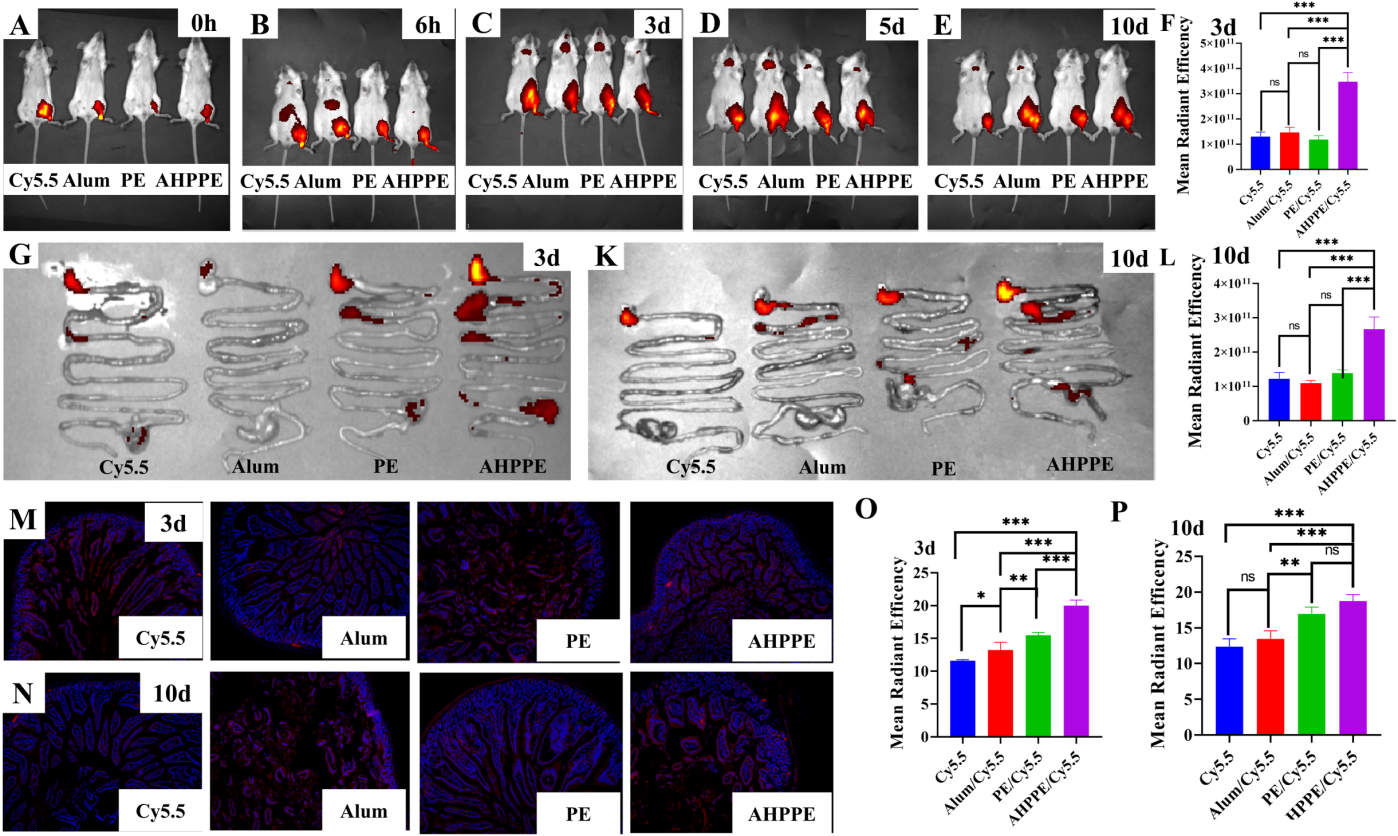
Fluorescence section diagram of AHPPE. **(A-E)***In vivo* fluorescence imaging of mice: **(A)** 0 days, **(B)** 6 hours, **(C)** 3 days, **(D)** 5 days, and **(E)** 10 days. **(G-K)** Fluorescence images of the mouse intestinal tract at various time points: **(G)** 3 days, and **(K)** 10 days. **(F)** 3 days fluorescence data, and **(L)** 10 days fluorescence data. **(M, N)** Fluorescence sections are utilized for analyzing drug targeting, where DAPI labels nuclei (blue dots) and Cy5.5 labels the drugs. The Cy5.5 fluorescence ratios are presented at different time points: **(M)** 3 days, and **(N)** 10 days. **(O)** 3 days fluorescence data, and **(P)** 10 days fluorescence data. Data presentation is in mean ± SEM format (n = 4). *P < 0.05, **P < 0.01, ***P < 0.001, ns, not significant.

To evaluate whether this adjuvant can target the intestine, *in vivo* fluorescence imaging was performed based on the aforementioned analysis of cell recruitment. The AHPPE emulsion exhibited detectable fluorescence in the intestine on day 3 post-injection. AHPPE emulsion maintained sustained high fluorescence levels in the intestinal tract at the 10-day time point (**Figures 3A–G**). The fluorescence signal from AHPPE adjuvant in the intestine remained significantly higher on days 3 and 10 compared to that observed in the PE, Alum, and the Cy5.5 groups *(P* < 0.05) (**Figures 3H–K**).

### 3.4 Expression of specific antibodies

**Figure 4:**
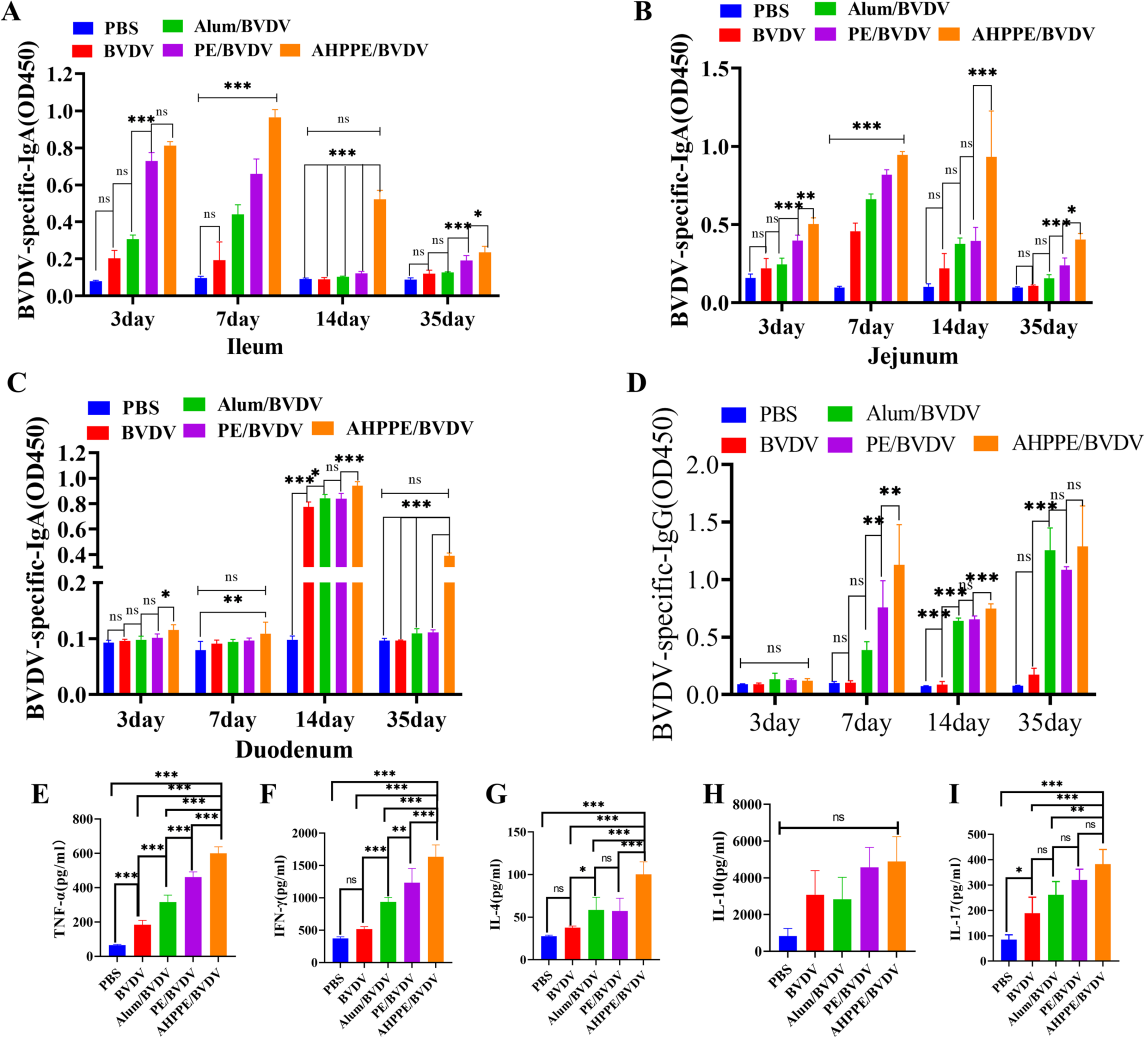
Serum levels of BVDV-specific antibodies. **(A)** BVDV-specific IgA antibodies in the ileum, **(B)** BVDV-specific IgA antibodies in the duodenum, **(C)** BVDV-specific IgA antibodies in the jejunum. **(D)** The levels of BVDV-IgG antibodies, Cytokines secretion level of **(E)** TNF-α, **(F)** IFN-γ, **(G)** IL-17, **(H)** IL-4, and **(I)** IL-10. Data presentation is in mean ± SEM format (n = 5). *P < 0.05, **P < 0.01, ***P < 0.001, ns, not significant.

The expression levels of specific antibodies were quantified by ELISA to assess the immunological efficacy of this adjuvant. Additionally, the AHPPE/BVDV group exhibited markedly elevated levels of BVDV-specific IgA in the duodenum, jejunum, and ileum on days 3, 7, 14, and 35 post-inoculation compared to the BVDV-alone, Alum/BVDV, and PE/BVDV groups (*P* < 0.05)(**Figures 4A–C**). The serum levels of BVDV-IgG were significantly higher in the AHPPE/BVDV, PE/BVDV, and Alum/BVDV groups compared to the BVDV-alone and PBS groups from day 7 to 35 (*P* < 0.05) (**Figure 4D**). The levels of TNF-α, IFN-γ, IL-4, and IL-17 were significantly increased in the AHPPE/BVDV group compared with the PE/BVDV, Alum/BVDV, and BVDV groups (*P* < 0.05) (**Figures 4E–H**). Furthermore, IL-10 expression was also significantly higher in the AHPPE/BVDV group compared to all other groups, except the PE group (*P* < 0.05) (**Figure 4I**).

### 3.5 AHPPE increases the number of IgA^+^ cells

**Figure 5:**
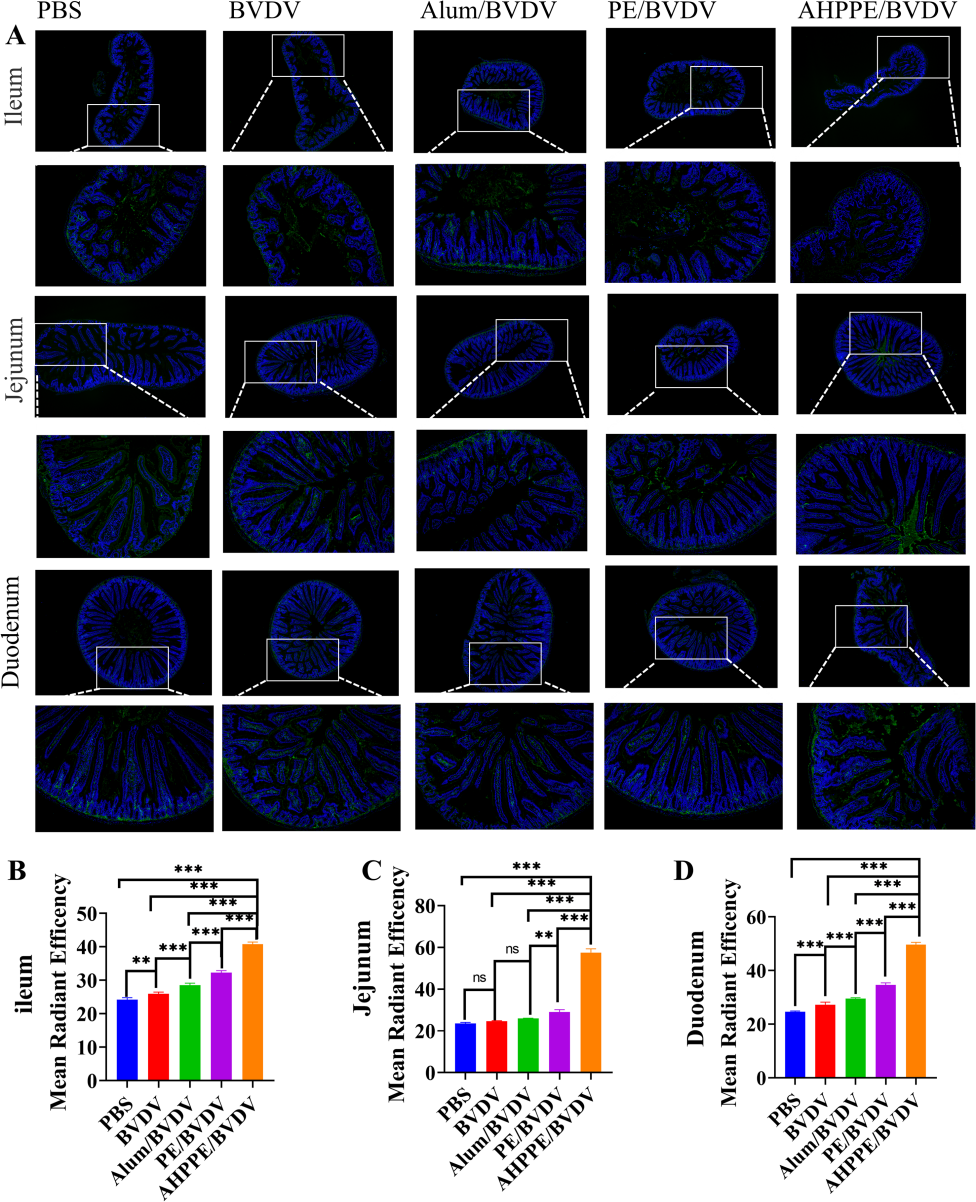
IgA expression analysis. **(A)** Immunohistochemical evaluation of IgA^+^ cell levels, with FITC labelling for IgA^+^ cells (green) and DAPI staining for nuclei (blue). Proportion of IgA^+^ cells in the **(B)** ileum, **(C)** jejunum, and **(D)** duodenum. Data presentation is in mean ± SEM format (n = 4). *P < 0.05, **P < 0.01, ***P < 0.001, ns, not significant.

To evaluate the intestinal targeting efficacy of AHPPE and its ability to elicit mucosal immune responses, the activation of IgA^+^ cells was assessed in the jejunum, ileum, and duodenum. As shown in **Figures 5A–E**, stimulation with AHPPE/BVDV significantly increased the proportion of IgA^+^ cells in all three intestinal segments compared to the other groups (*P* < 0.05). In particular, the highest proportion of IgA^+^-producing cells was observed in the jejunum.

### 3.6 Activation of CD4^+^ and CD8a^+^ T cells

**Figure 6:**
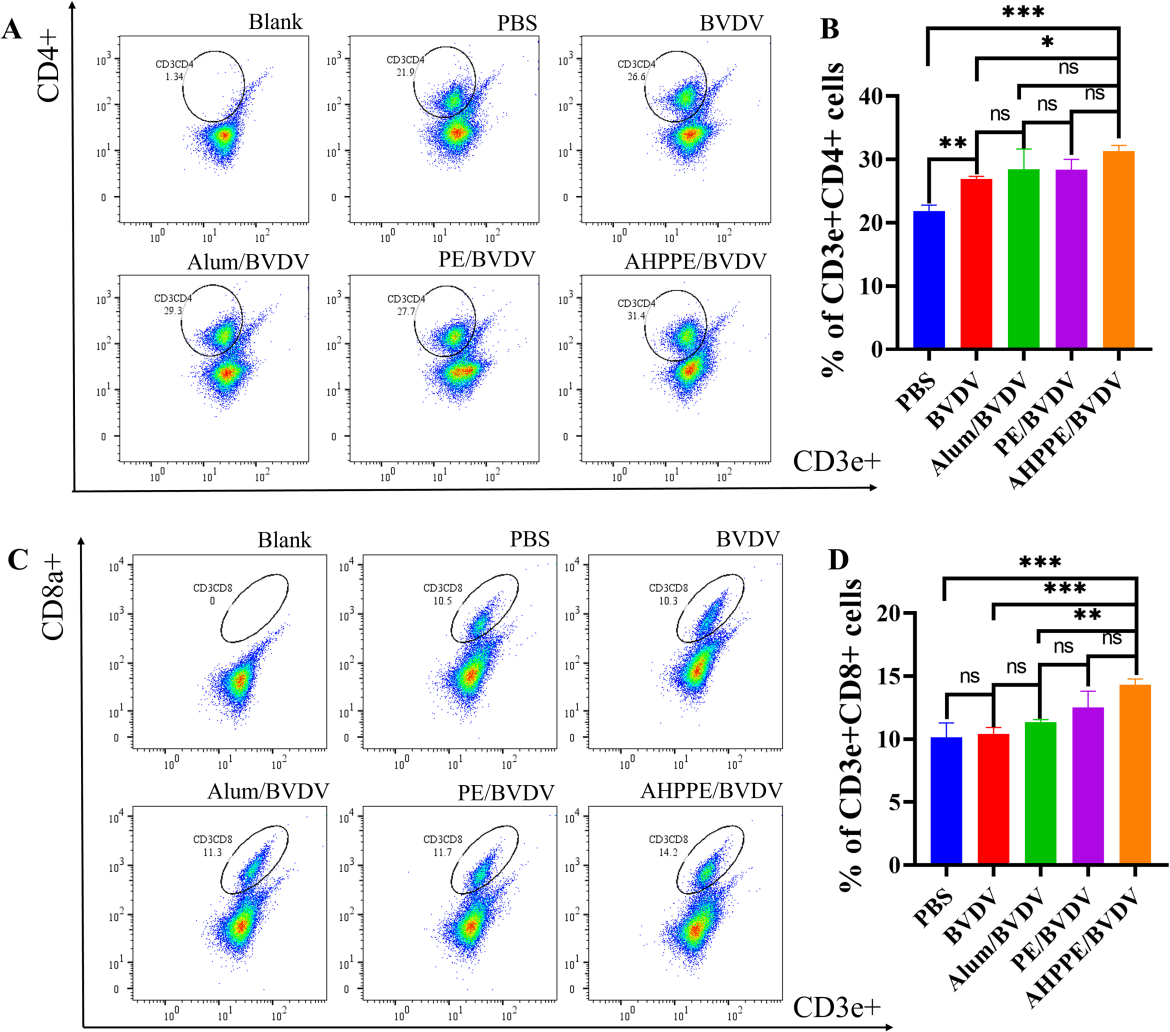
T-cell activation as measured by flow cytometry. Flow cytometry of the percentages of **(A, C)** CD3e^+^CD8a^+^ cells and **(B, D)** CD3e^+^CD4^+^ cells. Data presentation is in X ± SEM format (n = 4). *P < 0.05, **P < 0.01, ***P < 0.001, ns, not significant.

As shown in **Figures 6A, B**, the production of CD3e^+^CD4^+^ T cells was significantly increased in the AHPPE/BVDV group in contrast to all other groups (*P* < 0.05). Similarly, as shown in **Figures 6C, D**, the CD3e^+^CD8^+^ T cell population was significantly higher in the AHPPE/BVDV group compared to the BVDV group (*P* < 0.05).

### 3.7 Transcriptomic sequencing results

**Figure 7:**
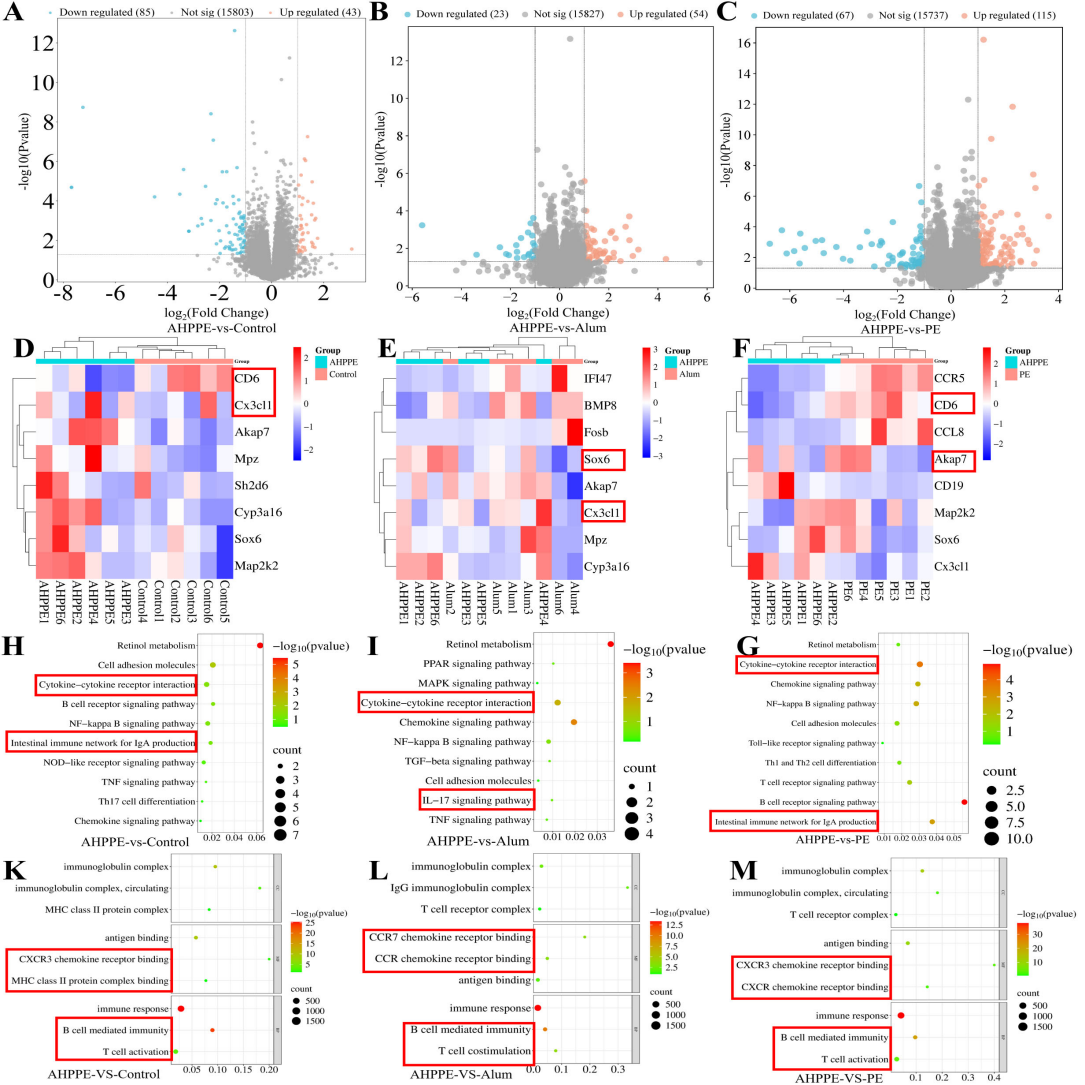
Transcriptome sequencing. **(A-C)** Differential genes expression analysis. **(D-F)** Differential Gene Expression Heatmap Analysis. **(H-I)** KEGG Enrichment Bubble Plot. **(K-M)** GO Enrichment Bubble Plot.

As shown in **Figures 7A–C**, compared with the AHPPE/BVDV group, the Control, Alum/BVDV, and PE/BVDV groups exhibited enrichment of 15,803, 15,827, and 15,737 genes, respectively. Among these enriched genes, 85, 23, and 67 were upregulated, while 43, 54, and 115 were down regulated in the respective groups. As shown in **Figures 7D–F**, Compared to the Control group, the up-regulated genes in the AHPPE/BVDV group were *Akap7, Sox6, Map2k2*, and *Cyp3a16*, while the down-regulated genes were *Cx3cl1*, *Cd6*, *Sh2d6*, and *Mpz*. Compared to the Alum/BVDV group, the upregulated genes in the AHPPE/BVDV group included *Akap7*, *Sox6*, *Cx3cl1*, and *Cyp3a16*, while the downregulated genes were *Mpz, Fosb, IFI47*, and *BMP8*. Compared to the PE/BVDV group, the upregulated genes in the AHPPE/BVDV group were *Akap7, Sox6, Map2k2, Cx3cl1*, and *CD19*, while the downregulated genes were of *CCR5, CCL8*, and *CD6*. KEGG enrichment analysis revealed enrichment of the Intestinal immune network for IgA production and B cell receptor signaling pathway in the AHPPE/BVDV group, which is associated with promoting IgA production. GO enrichment analysis further indicated that the MHC class II protein complex and B cell mediated immunity was enriched in the AHPPE/BVDV group, suggesting its involvement in immune response regulation.

### 3.8 Intestinal mRNA and protein expression levels

**Figure 8:**
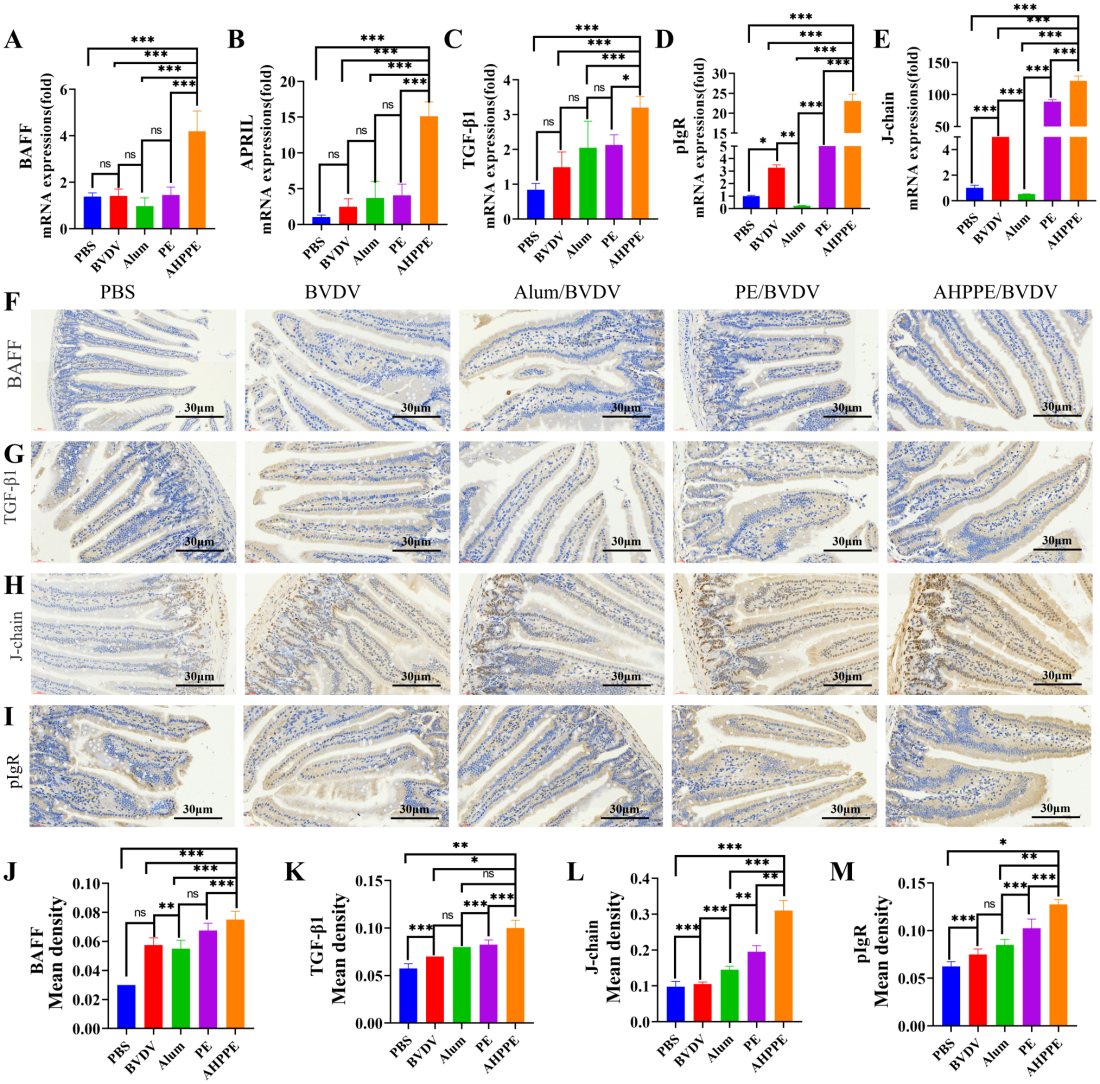
mRNA and protein expression levels. **(A-E)** The mRNA expression levels of BAFF, APRIL, TGF-β1, pIgR, and J-chain. **(H-M)** Protein levels detected by immunohistochemistry for BAFF, TGF-β1, APRIL, pIgR, and J-chain. Scale bar = 30 µm. Data presentation is in mean ± SEM format (n = 4). *P < 0.05, **P < 0.01, ***P < 0.001, ns: not significant.

KEGG enrichment analysis revealed enrichment in the Intestinal immune network for IgA production, suggesting that AHPPE may promote IgA generation via this pathway. We therefore performed targeted validation of this pathway. As can be seen in **Figures 8A–E**, the relative mRNA expression levels of *BAFF, APRIL, TGF-β1, J-chain*, and *pIgR* were significantly higher in the AHPPE/BVDV group compared to those in the other groups (*P* < 0.05). Immunohistochemical analysis further confirmed that the protein expression levels of *BAFF, TGF-β1, J-chain*, and *pIgR* were notably higher in the AHPPE/BVDV group relative to the control groups (**Figures 8D–F**, P < 0.05).

## 4 Discussion

Unlike traditional emulsions that depend on surfactants for stability, Pickering emulsions utilize the spontaneous adsorption of colloidal particles at the oil-water interface to achieve and maintain emulsion stability (22). While retaining the basic properties of conventional emulsions, Pickering emulsions offer several distinct advantages, including enhanced biocompatibility, outstanding resistance to aggregation, long-term physical stability, and high experimental reproducibility (23).

Pickering emulsions possess a surface morphology resembling that of a strawberry, enabling them to adsorb substantial quantities of antigens on their surfaces. **Figures 1F–I** in the present study demonstrates that the Pickering emulsion exhibits a raspberry-like morphology with abundant Dextran-FITC localized on the external surface, indicating high adsorption efficiency of AHPPE antigen. These observations are consistent with the findings of the study, thereby further validating the rationality of the fabricated nanomaterial. In terms of stability, both particle size and the uniformity of particle distribution play pivotal roles (22, 24). Previous studies have shown that nanoparticle systems with a narrow and uniform particle size distribution tend to be more stable, as they reduce inter-particle interaction and the likelihood of aggregation (25). The dynamic light scattering (DLS) technique provides a quantitative measure of particle size distribution, which is critical for evaluating nanoparticle stability. PDI is a critical parameter for assessing the stability of nanoparticles, with lower PDI values indicating a more homogeneous and stable system (26). **Figures 1J–Q** in this study shows that AHPPE has no sign of stratification and a uniform particle size, indicating excellent stability, which is consistent with the opposite conclusion and further verifies the stability of the nanomaterials. In this study, AHPPE cannot be freeze-dried, thus it has limitations in preservation and transportation, which is not conducive to large-scale application and promotion.

Additionally, we investigated the antigen-presenting cell recruitment and intestinal targeting capabilities of AHPPE *in vivo*, the recruitment of antigen-presenting cells CD11b^+^ and CD11c^+^ and the fluorescence intensity of intestinal nanoparticles were examined by flow cytometry and fluorescence sectioning. In a study, researchers have demonstrated that Pickering emulsions that significantly enhance vaccine efficacy by modulating antigen retention time and improving contact efficiency with APCs (18). Therefore, the CD11b is highly expressed in monocytes, macrophages, and immature DCs and plays a key role in cell migration (27). Contrary, CD11c is a specific marker for DCs, that are responsible for the cross-presentation of antigen to CD8^+^ T cells (28). In this study, the AHPPE/BVDV formulation significantly elevated the proportions of CD11c+ and CD11b^+^ cells (**Figure 2**). These results indicated that AHPPE is efficiently phagocytosed by dendritic cells and macrophages, so facilitating the targeted delivery of antigens to the gut. Therefore, these findings demonstrated that AHPPE was capable of recruiting a substantial number of DCs and macrophages at the injection spot.

In terms of immune regulation, Fan et al. (18) demonstrated that the Pickering emulsion prepared with Alum-loaded Polysaccharides from Poria cocos could modulate immune responses by promoting cytokine secretion, but its effect on mucosal immunity remains unclear. IgG serves as a key indicator of systemic responses, particularly those associated with Th2-type immunity (29). In the contradictory, IgA is predominantly localized in mucosal tissues. Deng et al. research found that after the injection of the agonist, a relatively high level of sIgA could be produced in the vaginal, intestinal and gastric mucosa, especially in the gastrointestinal tract (30). The results of this study are similar, all of which can induce high levels of sIgA in the intestinal tract; this study focuses on the local response in the intestinal tract and does not conduct related tests on other organs. It serves as the primary line of defense against pathogen invasion and exhibits functions such as immune regulation (31). Th1 cells play a crucial role in the induction of cellular immunity by activating macrophages through the secretion of cytokines, such as IFN-γ and TNF-α. They also facilitate the proliferation and differentiation of cytotoxic CD8^+^ T cells (32). Moreover, Th2 cells mediate humoral immunity by secreting cytokines such as IL-4 and IL-10, and by activating B cells to promote class switching of antibodies (especially IgE and IgG1) and support plasma cell differentiation (33). The CD3, as a core signaling component of the T-cell receptor (TCR) complex, synergistically plays a central role in antigen recognition, cellular activation, and effector functions with CD4 or CD8 (34). Through the systematic experiments shown in **Figures 4**-**6**, this study not only confirmed the regulatory effects of AHPPE on humoral and cellular immunity, but also supplemented its regulatory effects on mucosal immunity and the balance of T cell subsets through IGA fluorescence sections and flow cytometry analysis, thereby enriching the current understanding of the immune regulatory properties of Pickering emulsions.

Transcriptomics technology holds significant potential for enhancing our understanding of biological processes and mechanisms of action. KEGG enrichment analysis revealed that, compared with the PE and Control groups (**Figure 7E**), the AHPPE group exhibited significant alterations in metabolic pathways related to the intestinal immune network associated with Intestinal immune network for IgA production and the T cell receptor signaling pathway. The increase in IgA production mediated by Intestinal immune network for IgA production was consistent with the ELISA results (**Figure 3A**), further supporting that the AHPPE group significantly enhanced IgA secretion, thereby strengthening the body’s defense against enteroviruses. In contrast, the Alum group showed no changes in these metabolic pathways. GO enrichment analysis indicated that differentially expressed proteins were enriched in biological processes such as immune response, signal transduction, and the MHC class II protein complex. These findings suggest that AHPPE, as an immunoadjuvant, may enhance host immunity by Intestinal immune network for IgA production for IgA production, offering valuable insights into its potential immunomodulatory mechanisms. The production of intestinal IgA is primarily regulated by the Intestinal immune network for IgA production, IgA is abundantly expressed in the small intestine and plays a crucial role in maintaining intestinal immune function (35). The synthesis of secretory sIgA occurs through both T-cell-dependent and T-cell-independent pathways, with *TGF-β1* acting as a key regulator in the T-cell-dependent pathway (35–37). The relative gene and protein expression levels of *BAFF, APRIL*, and *TGF-β1* were significantly higher in the AHPPE/BVDV group compared to the other groups. These findings indicate that sIgA production can occur through both T-cell-independent and T-cell-dependent pathways (38). In the T-cell-independent pathway, sIgA can promote IgA class conversion by increasing the expression of *BAFF* and *APRIL* (37). The J-chain, acting as a ligand for the *pIgR*, binds to *pIgR* to initiate cellular transcytosis (39–41). During this process, the J-chain associates with IgA to form secretory sIgA, thereby enhancing mucosal immunity. The relative gene and protein expression levels of *pIgR* and *J-chain* were significantly higher in the AHPPE/BVDV group compared with the other groups. Collectively, these findings suggest that AHPPE/BVDV promotes IgA class switching through both T-cell-dependent and T-cell-independent pathways, thereby strengthening intestinal mucosal immune responses.

Previous studies have shown that Alum can up-regulate the expression of CD40L on the surface of B cells in mesenteric lymph nodes (mLNs), and in combination with IL-4 secreted by T cells, it can induce B cells to differentiate into IgA^+^ plasma cells, thereby increasing the level of intestinal sIgA (19). RA can promote IgA^+^ plasma cells to express α4β7 integrin, enabling them to migrate directionally to the intestinal mucosa and secrete sIgA. sIgA can bind to pathogens in the intestine, prevent them from adhering to epithelial cells, and strengthen the intestinal “immune barrier” (25). AHP can increase the number of intraepithelial lymphocytes (IELs) and IgA plasma cells in the lamina propria of the small intestine, enhancing the “first line of defense” of the intestinal mucosa (42). The combined effect of the three can ultimately build a protective intestinal immune system, while addressing the limitations of their individual use. At the same time, this study also has conclusions consistent with existing research, such as IgA plasma cells in the lamina propria of the small intestine, which further confirms the reliability and scientific nature of the experimental results of this study.

## 5 Conclusions

In this study, a stable AHPPE emulsion with high antigen-loading efficiency was successfully developed. *In vivo* evaluation demonstrated that the AHPPE emulsion exhibited strong intestinal targeting and sustained antigen release. Moreover, AHPPE induced robust and durable antibody responses and promoted the activation of CD4^+^ and CD8^+^ T cells, along with the secretion of associated cytokines. Additionally, the AHPPE emulsion enhanced the production of antigen-specific IgA by activating the intestinal immune network for IgA production and stimulating effective IgA secretion in the gut. Collectively, our findings highlight the potential of the AHPPE emulsion as a promising mucosal adjuvant capable of eliciting strong antigen-specific IgG and IgA responses through intramuscular immunization, thereby underscoring its value as an effective adjuvant for the prevention and control of infectious intestinal mucosal diseases.

## Data Availability

The datasets presented in this study can be found in online repositories. The names of the repository/repositories and accession number(s) can be found in the article/**Supplementary Material**.
