# Supplementary material for: Preparation and mechanism study of Alhagi honey polysaccharide-aluminum Pickering emulsion adjuvant for improving intestinal mucosal immune function
Source: Front Immunol. 2026 Feb 24;17:1775276. doi: 10.3389/fimmu.2026.1775276 (PMC12971699; doi:10.3389/fimmu.2026.1775276)
Supplement: Supplementary file 1 [file DataSheet1.docx]

**Table A1.** primer sequence

| **Gene** | 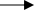**Primer sequence（5’-3’）** | **Base number** |
| --- | --- | --- |
| BAFF | F: TCCAGCAGTTTCACAGCGAT | 20 |
|  | R: GCAGGAATTGTTGGGCAGTG | 20 |
| APRIL | F: GCCCTTTCGGTTGCTCTTTG | 20 |
|  | R: TAGGCACGGTCAGGATCAGA | 20 |
| TGF-β1 | F: CCGCAACAACGCCATCTATG | 20 |
|  | R: GGATCCACTTCCAACCCAGG | 20 |
| PIgR | F: CTTCGGCAACCTGGAGTTCT | 20 |
|  | R: TGCTGTCTTTGGTCTTTTCT | 20 |
| J-chain | F: ACGACGAAGCGACCATTCTT | 20 |
|  | R: AGCAAGAATCGGGGGTCAAG | 20 |
| GAPDH | F: CCTCGTCCCGTAGACAAAATG | 20 |
|  | R: TGAGGTCAATGAAGGGGTCGT | 20 |


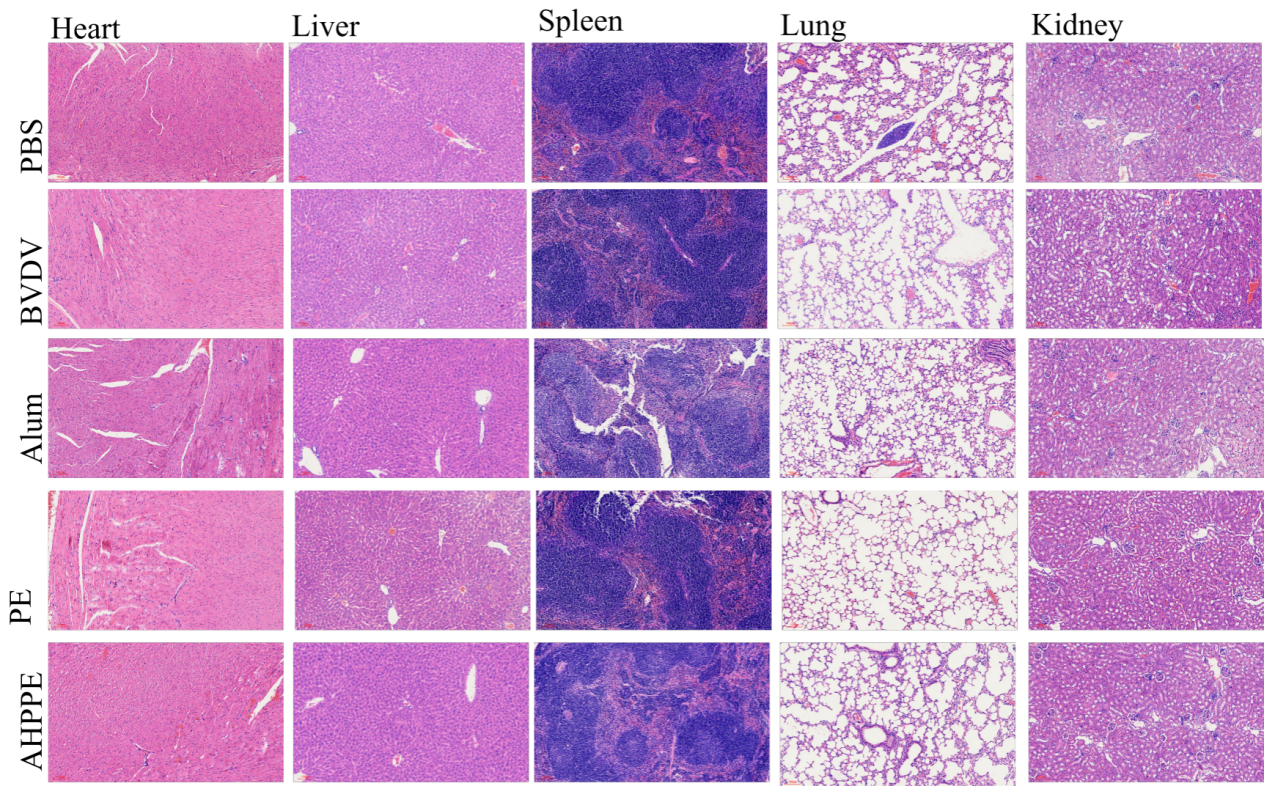


Figure S1 Safety Slice
